# Treatment of giant cell tumor of bone: Current concepts

**DOI:** 10.4103/0019-5413.32039

**Published:** 2007

**Authors:** Ajay Puri, Manish Agarwal

**Affiliations:** Dept. of Orthopedic Oncology, Tata Memorial Hospital, Mumbai, India

**Keywords:** Curettage, giant cell tumor, treatment

## Abstract

Giant cell tumor (GCT) of bone though one of the commonest bone tumors encountered by an orthopedic surgeon continues to intrigue treating surgeons. Usually benign, they are locally aggressive and may occasionally undergo malignant transformation. The surgeon needs to strike a balance during treatment between reducing the incidence of local recurrence while preserving maximal function.

Differing opinions pertaining to the use of adjuvants for extension of curettage, the relative role of bone graft or cement to pack the defect and the management of recurrent lesions are some of the issues that offer topics for eternal debate.

Current literature suggests that intralesional curettage strikes the best balance between controlling disease and preserving optimum function in the majority of the cases though there may be occasions where the extent of the disease mandates resection to ensure adequate disease clearance.

An accompanying treatment algorithm helps outline the management strategy in GCT.

Giant cell tumor (GCT) of bone is one of the commonest benign bone tumors encountered by an orthopedic surgeon. The reported incidence of GCT in the Oriental and Asian population is higher than that in the Caucasian population and may account for 20% of all skeletal neoplasms.^1^,^2^ It has a well-known propensity for local recurrence after surgical treatment.

Current recurrence rates between 10-20% with meticulous curettage and extension of tumor removal using mechanized burrs and adjuvant therapy are a vast improvement on the historically reported recurrence rates of 50-60% with curettage alone.

Certain controversies in the treatment of GCT continue to intrigue treating surgeons. Do adjuvants like phenol or cryotherapy for extension of curettage have any benefit; is it better to pack the defect with bone graft or cement; should a recurrent lesion be curetted again or widely excised; does one contemplate joint salvage or resection especially in large GCTs? These are some of the issues that offer topics for eternal debate.

This article endeavors to outline the principles of management of giant cell tumor of bone and addresses current opinion regarding some of these dilemmas.

## TREATMENT

The treatment of GCT is directed towards local control without sacrificing joint function. This has traditionally been achieved by intralesional curettage with autograft reconstruction by packing the cavity of the excised tumor with morsellised iliac cortico-cancellous bone. Regardless of how thoroughly performed, intralesional excision leaves microscopic disease in the bone and hence has a reported recurrence rate as high as 60%.^3^ Although a marginal or wide excision of the involved bone is curative if contamination is avoided, it is associated with reconstruction and disability problems. In order to counter the above problems, a great deal of effort has been expended on attempting to “extend” the curettage or intralesional excision by chemical or physical means.

### Intralesional curettage

The key to ensuring an adequate curettage with complete removal of tumor is obtaining adequate exposure of the lesion. This is achieved by making a large cortical window to access the tumor so as to avoid having to curette under overhanging shelves or ridges of bone. Use of a head lamp and dental mirror combined with multiple angled curettes helps to identify and access small pockets of residual disease which may otherwise result in recurrence. A high power burr to break the bony ridges helps extend the curettage and is recommended. A pulsatile jet lavage system used at the end of the curettage helps to bare raw cancellous bone and physically wash out tumor cells.

### Use of additional adjuvants to augment curettage

Adjuvants such as phenol used in a percentage varying from 5-80% after completion of curettage may be of additional benefit in helping to decrease recurrence rates after curettage.^4^ *In vitro* studies have also demonstrated the efficacy of using hydrogen peroxide as adjuvant therapy after extended local curettage for benign giant cell tumors of bone.^5^

**Figure 1:**
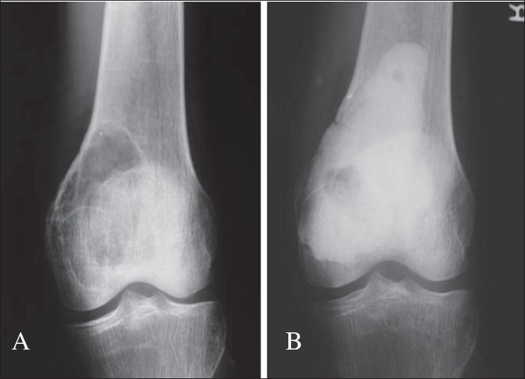
A) A.P. X-ray of a case of GCT lower end of femur, B) Treated with ‘extended intralesional curettage” and cementation

Cementation using methylmethacrylate has shown encouraging results^6^ [Figure 1]. It is postulated that the exothermic reaction of methylmethacrylate generates local hyperthermia which induces necrosis of any remaining neoplastic tissue, yet it does not extend to the normal tissues to result in local complications.^7^ In theory, the possibility that the polymerization of methylmethacrylate may produce a local chemical cytotoxic effect cannot be excluded. Cytotoxic agents like methotrexate and adriamycin have been incorporated in bone cement and other drug delivery systems in an attempt to reduce recurrence.^8^,^9^ Even pathological fractures through a giant cell tumor are not a contraindication to treatment by curettage and cementation.^10^,^11^ Cryosurgery using liquid nitrogen first propagated by Marcove, though used in some centers, is associated with a high incidence of local wound and bone complications.^12^,^13^

### Do These Adjuvants Help?

Some recent studies though, have questioned the role of adjuvants and filling agents in reducing the recurrence rate of giant cell tumors. Adequate removal of the tumor seems to be a more important predictive factor for the outcome of surgery than the use of adjuvants. The study by Trieb *et al* demonstrated that local recurrence rate of giant cell tumors located in long bones treated with or without phenol is similar.[14] Prosser *et al* retrospectively reviewed 193 patients treated during a 27-year period and compared their results with historic controls. One hundred and thirty-seven patients had curettage as a primary treatment and of these, 26 (19%) had local recurrences. The local recurrence rate of giant cell tumors confined to bone (Campanacci Grades I and II) was only 7% compared with 29% in tumors with extraosseous extension (Campanacci Grade III). They recommended primary curettage for intraosseous giant cell tumors without adjuvant treatment or filling agents, but tumors with soft tissue extension or with local recurrence may require more aggressive treatment.^15^

### Reconstructing the residual defect

Reconstructing the defect after curettage can be quite challenging. In case the gap left behind after the curettage is small and does not jeopardize the structural integrity of the bone it can be left alone and the cavities fill up with blood clot which then gets ossified to form bone.^15^ For larger defects the traditional methods of reconstruction have been cementation or use of bone graft with each method having its advantages and disadvantages.

### Advantages of bone graft

- Undergoes remodeling along stress lines
- Once incorporated, reconstruction is permanent

### Drawbacks of bone graft

- Autograft quantity is limited
- Donor site morbidity
- Allograft is expensive - requires a bone bank
- Recurrence relatively difficult to spot

### Advantages of cementing

- Methylmethacrylate monomer is cytotoxic
- Thermal effect - hyperthermia may help extend the boundary of tumor kill
- Radiographic detection of recurrence is easier
- Immediate structural support and rapid weight-bearing ambulation

### Drawbacks of cementing

- Not a biological material. Cement though strong in compression is relatively weak when subjected to shear and torsional forces. Hence its use in lesions involving the head and neck of the femur may result in an increased chance of fractures through cement.
- Fear about long-term degeneration of articular cartilage in subchondral lesions in weight-bearing areas

Recent studies have demonstrated the efficacy of bone substitutes like calcium phosphate as a filling agent.^16^ If the patient is treated by cementation, there is a belief that it is necessary to remove the cement after an appropriate passage of time (to be reasonably certain that local relapse is not going to develop). The defect is subsequently reconstructed with autograft on the subchondral portion of the repair supplemented with allograft to prevent late articular degeneration. However, studies have shown that joint function is not compromised in time even after the use of subchondral cement.^17^,^18^ There is an interesting report on two cases by Tejwani *et al*, both with symptomatic full-thickness tibial articular cartilage loss and one with a meniscal tear, after curettage, phenol cautery and PMMA reconstruction of giant cell tumor of the proximal tibia. Arthroscopic chondroplasty and planing of the exposed cement was performed in both cases, theoretically reducing focal areas of stress concentration that could lead to further meniscal damage and injury to the femoral condyle articular surface in weight-bearing.^19^

**Figure 2:**
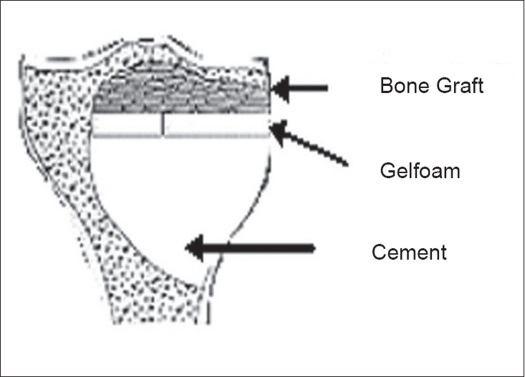
Diagrammatic representation of reconstruction of GCT with minimal subchondral bone

**Figure 3:**
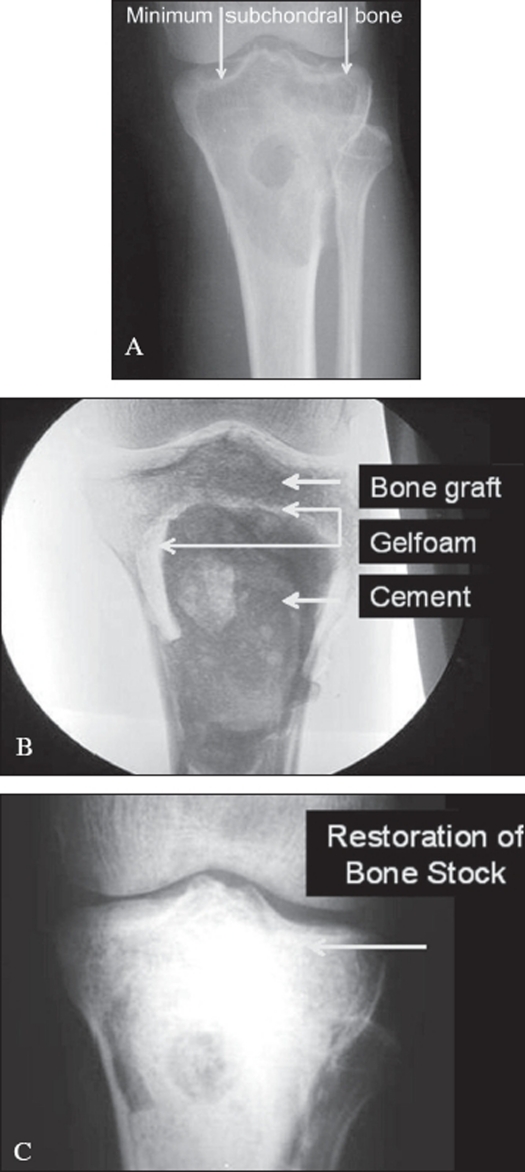
A), B), C): Reconstruction of GCT with minimal subchondral bone

To try and forestall this potential problem of late articular degeneration in subarticular lesions where the amount of residual subchondral bone after an extended curettage is less than 5 mm, a multilayer reconstruction technique is recommended. A mixture of morsellized auto and allograft (about 5-8 mm thick) is packed adjacent to the subarticular surface. A layer of gelfoam is layered over this and the remaining cavity is packed with cement [Figure 2]. This helps reduce heat damage from the curing cement, and the subarticular bone graft after consolidation should theoretically prevent articular degeneration.^20^ Another perceived advantage is that should recurrence occur, the danger of damage to articular cartilage during removal of cement is reduced [Figure 3].

Occasionally, Steinmann pins have been used to reinforce the bone cement used to fill the large subchondral defects following intralesional curettage. However, whether this is of real benefit in improving the stability of the defect is controversial.^21^ Large lesions can cause weakening of the structural stability of bone. Depending on the residual structural integrity of the host bone it may be necessary to augment the construct with internal fixation.

### Wide resection and subsequent reconstruction

**Figure 4:**
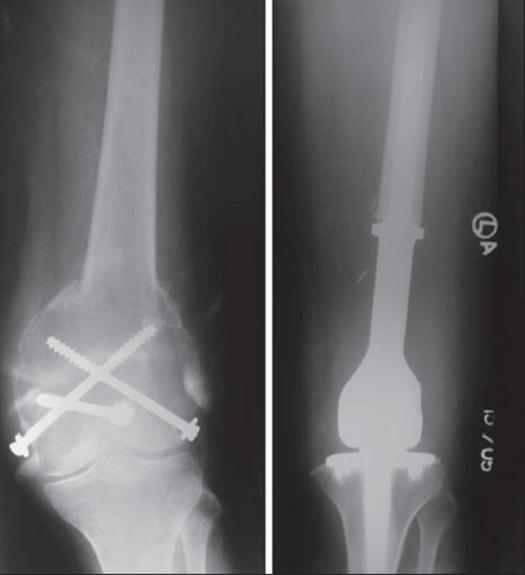
Large recurrent GCT with pathological fracture, treated with resection and megaprosthesis

In a study of 38 patients with giant cell tumor in the knee region Chen *et al* measured the area of affected subchondral bone radiographically using plain radiographs, CT and MRI and correlated it with the mean Enneking functional score at follow-up. In patients initially treated with curettage and bone grafting, the mean area of initially affected subchondral bone was 18.6% with a linear trend showing that the larger the area of affected subchondral bone, the worse the functional score. Among patients initially treated with wide resection, the mean area of affected subchondral bone was 68.2%.^20^ Thus occasionally, even in benign tumors, resection may be the preferred option when bone salvagibility by intralesional methods would result in such severe mechanical compromise that skeletal integrity is unlikely to be maintained or unlikely to be restored after healing, leading to a compromise in ultimate function^21^ [Figure 4]. In certain bones like the lower end ulna, upper end fibula etc. excision may be attempted as the treatment of choice.

If marginal / wide local excision is elected as the treatment of the lesion, either primarily or in recurrence, then reconstruction necessarily implies reconstruction of the joint surface, since GCT invariably involves the end of a long bone and causes significant dysfunction of the joint surface.^22^,^23^

### The options include

- Megaprosthetic joint replacement: These afford stability and mobility, however, are prone to ultimate loosening, wear or breakage and require revisions.
- Biologic reconstruction: These are technically demanding, but durable procedures affording stability at the cost of mobility. They include:
- autograft arthrodesis (knee, wrist, shoulder) with internal / external fixation^24^
- live microvascular fibula reconstructions (e.g., around knee and shoulder, distal radius reconstruction, distal fibula GCT with ankle reconstruction)^25^–^27^
- Ilizarov method of bone regeneration^28^,^29^
- osteo-articular allografts^30^,^31^ (complications include infection, nonunion, graft fracture and instability).

### Lower end radius lesions

**Figure 5:**
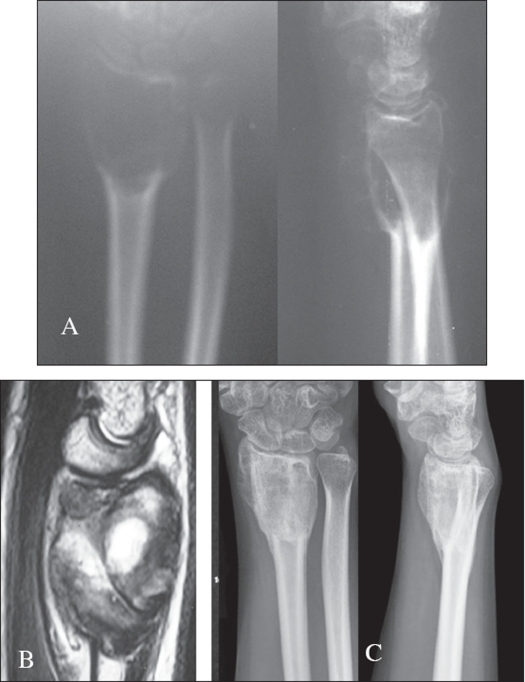
A) X-ray (A.P. and lateral), B) MRI of GCT of lower end radius, C) Two years followup X-ray of the same after intra lesional curettage and bone grafting showing healed lesion.

There is some debate regarding the management of GCT in the lower end radius. Some authors have reported a high rate of local recurrence in GCT of the distal radius and recommend that they should be treated more aggressively. Today the consensus of opinion would state that curettage should be attempted for the majority of patients with GCT of the distal radius [Figure 5] but some form of stabilization may be required in the presence of extensive bone destruction.^32^ Cheng *et al.* state that intralesional excision should not be excluded as a possible treatment of even Grade III lesions. They recommend Grade III lesions be treated with curettage when the tumor does not invade the wrist, destroy more than 50% of the cortex or break through the cortex with an extraosseous mass in more than one plane.^31^

## LOCAL RECURRENCE IN GCT

Local recurrences appear to be related to the surgical margin and are clinically characterized by pain and radiologically by progressive lysis of the bone graft or the adjacent cancellous bone. Following curettage and cementation an osteolytic zone caused by thermal injury measuring 2 mm surrounds the cement. This radiolucent zone is bordered by a thin outer sclerotic rim for about six months. Lysis or failed development of the sclerotic rim between the cement and cancellous bone may suggest recurrence.^33^ Soft tissue recurrence is visible on plain radiographs because of its tendency towards peripheral calcification. A recent study by Akhane *et al* suggests that total serum acid phosphatase (TACP) could be used as a tumor marker for monitoring response to the treatment of GCT. Total serum acid phosphatase level in GCT patients correlated with tumor size. The high preoperative TACP values in GCT patients became normalized after surgery but reappeared in three of five patients with local recurrence.^34^

Though the majority of recurrences usually occur within the first two years, late recurrences are known and long-term surveillance is recommended in these patients.^35^,^36^ Even though the increasing grade from I to III is not a reflection of the biologic aggressiveness of the tumor, various authors have documented an increased rate of recurrence in Grade III lesions.^15^,^18^ This could be due to the difficulty in achieving complete clearance once the tumor has breached its normal anatomic boundaries and extended into soft tissue.

The principles of management remain the same even in recurrent tumors. Steyern *et al.* retrospectively studied (n = 137) local recurrence of GCT in long bones following treatment with curettage and cementing. They concluded that local recurrence after curettage and cementing in long bones can generally be successfully treated with further curettage and cementing, with only a minor risk `of increased morbidity.^37^ This suggests that more extensive surgery for the primary tumor in an attempt to obtain wide margins is not the method of choice, since it leaves the patient with higher morbidity with no significant gain with respect to cure of the disease. Mcgough *et al.* retrospectively reviewed 183 consecutive patients diagnosed with GCT at the three most common sites (distal femur, proximal tibia and distal radius) to determine the pattern of local tumor recurrence and the impact severity of the recurrence on adjacent joint function. The primary tumor was treated in all patients with intralesional excision of tumor by curettage. Forty-five patients developed locally recurrent disease. They opined that meticulous attention to surgical detail and close postoperative surveillance for successful local tumor control and durable, joint-preserving function was necessary. Incomplete initial surgery, a delay in diagnosis of the recurrence of greater than six months and subchondral recurrence of tumor were contributing factors in the failure to salvage the joint.^38^

## CHEMOTHERAPY AND RADIOTHERAPY

Occasional GCT of bone demonstrate profound responses to chemotherapy but these cases are anecdotal and their incidence is disappointing. At the present time there are no recognized effective chemotherapeutic agents available for the management of these tumors. The literature documents a close association of secondary sarcomatous transformation in the region of GCTs treated by radiation therapy. Though surgery remains the treatment of choice, radiotherapy is recommended when complete excision or curettage is impractical for medical or functional reasons (generally for lesions of the spine and sacrum) or for aggressive, multiply recurrent tumors.^39^–^42^ In lesions involving the axial skeleton, with the exception of the sacrum, excision with stabilization of the spine and biologic reconstruction of the anterior column^43^ followed by reduced levels of irradiation (45 Gy in 4.5 weeks), on the assumption that you are dealing with microscopic residual tumor only, would offer the patient the best chance of long-term local control. The use of modern-day techniques and megavoltage radiation may help to reduce the rate of malignant transformation that was seen during the earlier era of orthovoltage radiation.^39^

## EMBOLIZATION

Unresectable GCTs (e.g., certain sacral and pelvic tumors) can be managed with transcatheter embolization of their blood supply. Since flow reconstitution invariably occurs, embolization is performed at monthly intervals until significant pain palliation is achieved. Subsequent embolizations are performed when there is symptomatic or radiographic relapse of the tumor.^3^,^44^ Tumors in areas amenable to surgical resection also benefit by preoperative embolization in an attempt to reduce the amount of intraoperative blood loss.

## BISPHOSPHONATES

Recent reports indicate that topical or systemic use of pamidronate or zoledronate can be a novel adjuvant therapy for giant cell tumor. Bisphosphonates act by targeting osteoclast-like giant cells inducing apoptosis and limiting tumor progression.^45^,^46^

A recent study has shown that rinsing of morcellized bone grafts with bisphosphonates prevents resorption and is likely to reduce the risk of mechanical failure. Though this was studied during revision total hip replacement using morcellized compacted bone allograft, the same principle may possibly be applicable to bone grafts used to fill defects after curettage.^47^

## METASTASIS IN GCTS

The incidence of metastases is estimated to be from 1-6%. The metastatic lesions are histologically identical to the primary lesions, showing no tendency to dedifferentiate. The majority of metastatic lesions are to the lung. Solitary metastasis to regional lymph nodes, the mediastinum and the pelvis have been reported, as has involvement of the scalp, bone and paraaortic nodes.^48^–^52^ The mean interval between the onset of the tumor and the detection of lung metastases is about four to five years. The natural history of metastatic lesions is unpredictable. Complete excision of metastases has been very successful with good long-term survival, but those with inoperable disease may die from metastases. Hence, metastatic lesions should be resected if possible. Radiation and chemotherapy have enjoyed limited success. Steroids have been successfully used in the control of unresectable metastases. Though rare, there are several reports where the metastases have completely regressed spontaneously or have remained static for years. There have been several reports of long-term survival even with residual pulmonary tumors. Metastatic disease in GCT does not carry the same poor prognosis as malignant tumors. Therapy should be directed at achieving adequate local control and if possible complete excision of the metastatic lesions.

## MALIGNANT GCT

There are mainly two kinds of malignant GCT. The primary malignant GCTs are rare where a giant cell tumor-like area exists alongside a high-grade sarcoma.^53^ The radiological features are sometimes difficult to differentiate from a benign GCT. These would more appropriately be termed as giant cell rich sarcomas and should be treated on similar lines as a primary bone sarcoma. Secondary malignant GCTs are more common. These include irradiated GCTs where a sarcomatous transformation has occurred. Sometimes a malignant sarcoma develops at the site of a previously treated GCT. In both these instances the sarcoma is either a high-grade osteosarcoma, MFH or fibrosarcoma. These have a poor prognosis, particularly the radiation-induced sarcomas.

**Figure 6:**
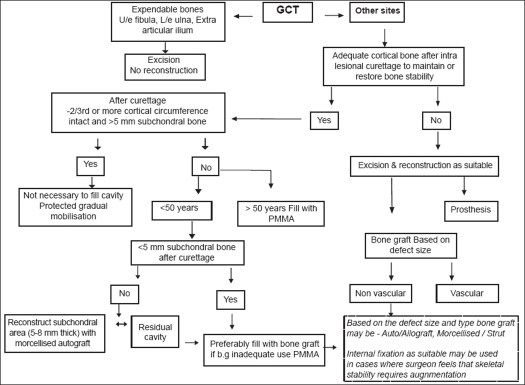
Treatment algorithm

GCT though benign is locally aggressive and the surgeon needs to strike a balance during treatment between reducing the incidence of local recurrence while preserving maximal function [Figure 6]. In 1912 Joseph Bloodgood was the first to refer to this lesion as “giant cell tumor”. His suggestions that this tumor was preferably treated by curettage with chemical cauterization and bone grafting are still widely followed.^54^ Current literature too suggests that intralesional curettage strikes the best balance between controlling disease and preserving optimum function in the majority of the cases though there may be occasions where extent of the disease mandates resection to ensure adequate disease clearance.

Between 10-20% of tumors would still recur in spite of our best efforts. The principles governing the management of recurrent tumors remain the same as it is believed that more extensive surgery in an attempt to obtain wider margins leaves the patient with higher morbidity with no significant gain with respect to cure of the disease.
